# Transdiagnostic Phenotyping Reveals a Host of Metacognitive Deficits Implicated in Compulsivity

**DOI:** 10.1038/s41598-020-59646-4

**Published:** 2020-02-19

**Authors:** Tricia X. F. Seow, Claire M. Gillan

**Affiliations:** 1 School of Psychology, Trinity College Dublin, Dublin, Dublin 2 Ireland; 2 Trinity College Institute of Neuroscience, Trinity College Dublin, Dublin, Dublin 2 Ireland; 3 Global Brain Health Institute, Trinity College Dublin, Dublin, Dublin 2 Ireland

**Keywords:** Decision, Obsessive compulsive disorder

## Abstract

Recent work suggests that obsessive-compulsive disorder (OCD) patients have a breakdown in the relationship between explicit beliefs (i.e. confidence about states) and updates to behaviour. The precise computations underlying this disconnection are unclear because case-control and transdiagnostic studies yield conflicting results. Here, a large online population sample (N = 437) completed a predictive inference task previously studied in the context of OCD. We tested if confidence, and its relationship to action and environmental evidence, were specifically associated with self-reported OCD symptoms or common to an array of psychiatric phenomena. We then investigated if a transdiagnostic approach would reveal a stronger and more specific match between metacognitive deficits and clinical phenotypes. Consistent with prior case-control work, we found that decreases in action-confidence coupling were associated with OCD symptoms, but also 5/8 of the other clinical phenotypes tested (8/8 with no correction applied). This non-specific pattern was explained by a single transdiagnostic symptom dimension characterized by compulsivity that was linked to inflated confidence and several deficits in utilizing evidence to update confidence. These data highlight the importance of metacognitive deficits for our understanding of compulsivity and underscore how transdiagnostic methods may prove a more powerful alternative over studies examining single disorders.

## Introduction

Intentional decisions are dependent on the interplay between behaviour and beliefs. Beliefs guide behaviour, and the consequences of our behaviour in turn update beliefs. Computational models of learning suggest that the strength of belief (i.e. “confidence”) governs the extent of its influence on action; the more confident we are, the more our behaviour is influenced by pre-existing beliefs, compared to new information^1,2^. A breakdown in the relationship between action and belief is suggested to be characteristic of compulsive behaviours, e.g. in obsessive-compulsive disorder (OCD) or addiction. In these disorders, behaviour often appears autonomous, unguided by conscious control and ‘ego-dystonic’, such as persistent drug use despite negative consequences^3^ or out-of-control repetitive checking despite knowing the door is locked^4^. One potential cause of the divergence between intention and action in compulsive individuals is an impairment in the brain’s goal-directed system, which links actions to consequences and protects against overreliance on rigid habits^5^. Goal-directed planning deficits have been consistently observed in OCD^6^^–^^9^ and related disorders^10^—there is evidence to suggest this constitutes a transdiagnostic psychiatric trait linked to several aspects of clinically-relevant compulsive behaviour^11^.

The precise mechanism supporting this dysfunction is only partially understood as most employed tasks struggle to separate the construction of an internal model (e.g. action-outcome knowledge) from its implementation in behaviour. Those that have attempted this have yielded interesting, if equivocal, results. One study showed that OCD patients get stuck in habits even when they possess the requisite action-outcome knowledge to theoretically perform in a goal-directed fashion^7^. This suggests that the implementation of goal-directed behaviour is deficient in OCD, independent of the ability to construct the model. However, this does not mean the internal model is intact; studies using more challenging tasks have found deficits in the acquisition of explicit action-outcome contingency knowledge in OCD patients^9^, suggesting that patients may have problems with both. However, these findings come from paradigms where instrumental action typically affects the kind of information that is gathered and thus are somewhat confounded and difficult to interpret. Recently Vaghi and colleagues addressed this metacognitive question in OCD patients with more precision by using a paradigm that examined how patients make trial-wise adjustments to behaviour (i.e. implicit model) and confidence (i.e. explicit model) in response to feedback^12,13^. They found that in OCD, the association between confidence and behavioural updating (‘action-confidence coupling’) was diminished—patients’ behaviour did not align with their internal model. Further, while confidence estimates did not differ from healthy controls, OCD patients showed abnormalities in their learning rate, making more trial-wise adjustments in response to feedback than controls^13^.

The finding of intact confidence in OCD is consistent with prior work in perceptual decision-making where individuals high versus low in OCD symptoms had no differences in their mean confidence^14^—results echoed by two large internet-based samples (N > 490) we conducted with the same task that also found no relationship to OCD symptoms^15^. A limitation of this type of study design, however, is that it fails to capture the potentially competing influence of co-occurring disorders/symptoms in psychiatric populations. Even in studies where certain co-morbid diagnoses are explicitly excluded for, as in Vaghi *et al*., rates of depression and anxiety are greater than controls^13^. Similarly, when self-report anxiety and depression severity are matched across groups by design, as in Hauser *et al*.^14^, this may not accurately reflect the average OCD patient where co-morbidity is the rule, not the exception (e.g. >25% of OCD patients are co-morbid for ≥4 additional diagnoses^16^). An alternative approach to resolving this isssue measures relevant co-occurring symptoms in the same individuals and seeks to account for their (deflating or inflating) influence on the cognitive measure of interest. We took this approach in a prior study and found that confidence abnormalities in perceptual decision-making were reliably associated with two transdiagnostic psychiatric dimensions in opposing directions: ‘anxious-depression’ was associated with reduced confidence, while ‘compulsive behaviour and intrusive thought’ was linked to inflated confidence^15^. This finding was striking because confidence was not correlated with either OCD or depressive symptoms in the same sample. Given this, it is possible that true metacognitive abnormalities in OCD were obscured by the competing influence of co-occurring depression in this dataset, and potentially, this same issue is at play in the prior case-control study examining metacognition in OCD in the context of reinforcement learning.

To test this, we used the same transdiagnostic methodology on an online sample of 437 participants who completed the task employed by Vaghi and colleagues^13^. We investigated the extent to which trial-wise action adjustments were disconnected from confidence reports in individuals high in self-reported OCD symptoms, and whether this action-confidence decoupling is specific to OCD or also manifests in other psychiatric phenomena. We then tested if transdiagnostic phenotyping would reveal a more specific result—that only the compulsive dimension (as opposed to anxious-depression and social withdrawal) would be related to the decoupling of confidence and behaviour. Lastly, we investigated if the decoupling is linked to alternations in action-updating or confidence, and, with the same reduced Bayesian model used in the original study^2,12,13,17^, tested if there were abnormalities in the way compulsive individuals used information (e.g. recent outcomes, unexpected outcomes, environmental uncertainty and positive feedback) to update behaviour and confidence.

## Methods

### Power estimation

Previous research utilizing the predictive-inference task were constrained to small sample psychiatric populations^13^. As such, we referred to earlier work that investigated confidence abnormalities in large general population cohorts with transdiagnostic symptom dimensions to determine an appropriate sample size^15^. The prior study reported an association of the ‘anxious-depression’ factor with lowered confidence level (*β* = −0.20, *p* < 0.001), an effect size suggesting that N = 295 participants were required to achieve 90% power at 0.001 significance level.

### Participants

Data were collected online using Amazon’s Mechanical Turk (N = 589). Participants were ≥18 years, based in USA and had >95% of their previous tasks on the platform approved. After reading the study information and consent pages, they provided informed consent by clicking the ‘I give my consent’ button. Participants were paid a base sum of 7 USD plus up to 1 USD bonus. Of the sample, 249 were female (42.3%) with ages ranging from 20–65 (mean = 36.3. SD = 10.2) years. All study procedures were approved by and carried out in accordance with regulations and guidelines of Trinity College Dublin School of Psychology Research Ethics Committee.

### Exclusion criteria

Several pre-defined exclusion criteria were applied to ensure data quality (see Supplemental Methods for exclusion criteria details). In total, 153 participants (25.9%) were excluded, a rate typical for web-based experiments, leaving 437 participants for analysis. Of this, N = 20 (4.58%) of the current sample were the same participants from experiment 1 of a prior study where we examined confidence in perceptual decision-making^15^. An additional 10 participants (2.29%) of the current sample were included from experiment 2 of that same paper.

### Predictive inference task

**Figure 1:**
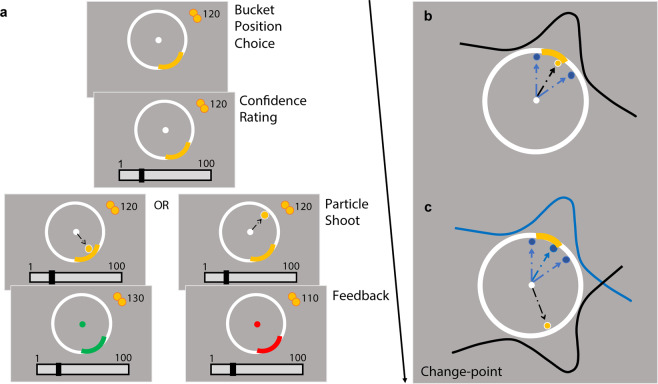
Predictive-inference task. (**a**) Trial sequence. Participants were instructed to position a bucket (yellow arc on the circle edge) to catch a flying particle, and thereafter rated their confidence that they would catch the particle. Particles were fired from the centre of the circle to the edge. Points were gained when the particle was caught, and the bucket turned green; else, points were lost and the bucket turned red. (**b**) Particle trajectories. For every trial, landing locations were independently sampled from a Gaussian distribution. As such, particles landed around the same area with small variations induced by noise. For illustration purposes, dashed arrow lines represent particle trajectory of current (black) and past (blue) trials, which over trials allow subjects to generate a representation of the Gaussian. (**c**) Change-points. The mean of the distribution abruptly moves to another point on the circle when a “change-point” occurs. This new mean is then sampled in the same manner as (**b**) until the next change-point.

We adapted the predictive-inference task from Vaghi *et al*.^13^ for web-based testing (Fig. 1). Left and right arrow keys enabled response navigation while a spacebar press was used for decision confirmation (this is in contrast to a rotor controller used for response navigation in Vaghi *et al*.). The aim of the task presented to participants was to catch a particle flying from the centre of a large circle to its edge. To do so, participants positioned a ‘bucket’ (a free-moving arc) on the circle edge at the start of each trial. Once the bucket location was chosen, a confidence bar scaling 1 to 100 would appear below the circle after 500 ms. The confidence indicator would begin randomly at either 25 or 75. Participants then indicated how confident they were that the particle would land in the bucket. After confirmation of the confidence report, a particle was then released from the centre to fly towards the edge of the circle 800 ms later. If the particle landed within the boundaries of the bucket, the bucket would turn green for 500 ms and the participant gained 10 points; else, the bucket turned red for 500 ms and lost 10 points. The number of points accumulated over the task was presented in the top right-hand corner for participants to track their performance. Payment was partially performance contingent; the more points earned, the higher amount of bonus they received at the end, up to a maximum of 1 USD on top of their flat fee of 7 USD. Confidence ratings were not incentivized.

On each trial, the particle’s landing location on the circle edge was sampled independently and identically from a Gaussian distribution with SD = 12. As such, the particle landed in the same location with small variations determined by noise. The mean of this distribution did not change until a change-point trial was reached, where it was re-sampled from a uniform distribution U(1,360) (i.e. the number of points on the circle). Participants would therefore have to learn the mean of the new generative distribution after a change-point. The probability of a change-point occurring on each trial was determined by the hazard rate. In the task, there were two hazard rate conditions that varied the number of change-points in a stretch of 150 trials each: stable (hazard rate = 0.025, 4 change-points), and volatile (hazard rate = 0.125, 19 change-points). Hazard rate conditions were not relevant to the analyses of the current paper. The order of hazard rate conditions was randomly shuffled, as were the order of change-points within a condition. Participants completed 300 trials in total, divided into 4 blocks of 75 trials, with no explicit indication when a change in condition block occurred. Breaks were given between blocks which did not fall before the switch of a new hazard rate condition.

Before the start of the task, participants were instructed on the aim of the experiment and shown its layout. Participants then completed 10 practice trials that were excluded from the analysis and did not count for their final score. After the practice, they had to answer 5 quiz questions pertaining to the task instructions. If they answered any of the questions wrong, they would be brought back to the beginning of the instructions and taken through the practice block again. Additionally, in order to reduce the number of participants failing to utilize the confidence scale properly, the task was reset to the beginning if participants left their confidence ratings as the default score for more than 70% of the trials at the 20th and 50th trial mark. They would have to complete the instruction quiz again before proceeding with the task.

### Self-report psychiatric questionnaires & IQ

Participants completed a range of self-report psychiatric assessments after the behavioural task. To enable application of the transdiagnostic analysis with psychiatric dimensions described in previous studies^11,15^, we administered the same nine questionnaires assessing: *Alcohol addiction* using the Alcohol Use Disorder Identification Test (AUDIT)^18^, *Apathy* using the Apathy Evaluation Scale (AES)^19^, *Depression* using the Self-Rating Depression Scale (SDS)^20^, *Eating disorders* using the Eating Attitudes Test (EAT-26)^21^, *Impulsivity* using the Barratt Impulsivity Scale (BIS-10)^22^, *Obsessive-compulsive disorder* (OCD) using the Obsessive-Compulsive Inventory – Revised (OCI-R)^23^, *Trait anxiety* using the trait portion of the State-Trait Anxiety Inventory (STAI)^24^, *Schizotypy* scores using the Short Scales for Measuring Schizotypy^25^, and *Social anxiety* using the Liebowitz Social Anxiety Scale (LSAS)^26^. Consistent with the original study that defined these transdiagnostic traits^11^, item 13 on the Self-Rated Depression Scale^20^ was erroneously administered as “I am restless and can’t sleep”, instead of “I am restless and can’t keep still”. The order of these self-report assessments administered was fully randomized. Following the questionnaires, participants completed a Computerized Adaptive Task (CAT) based on items similar to that of Raven’s Standard Progressive Matrices (SPM)^27^ to approximate Intelligence Quotient (IQ).

### Transdiagnostic factors (dimensions)

Raw scores on the 209 individual questions that subjects answered from the 9 questionnaires were transformed into factor scores (AD: ‘anxious-depression’, CIT: ‘compulsive behaviour and intrusive thought’, and SW: ‘social withdrawal’), based on weights derived from a larger previous study (N = 1413)^11^ (Supplementary Fig. S6). These factors are not orthogonal and therefore correlate moderately, with values ranging from *r* = 0.34–0.52.

### Action-confidence coupling

Regression analyses were conducted using mixed-effects models written in R, version 3.5.1 via RStudio version 1.1.463 (http://cran.us.r-project.org) with the *lme4* package. We examined the coupling between trial-by-trial action updates (*Action*, the absolute difference of bucket position on trial *t* and *t* + 1, as the dependent variable) and confidence (*Confidence*, confidence level on trial *t* + 1, z-scored within-participant, as the independent variable) with age, gender and IQ as z-scored fixed effects co-variates. Within-subject factors (the intercept and main effect of *Confidence*) were taken as random effects (i.e., allowed to vary across subjects). To test if questionnaire total scores or transdiagnostic dimension severities were associated to changes in action-confidence coupling, the scores were included as z-scored between-subjects predictors in the basic model above. Separate regressions were performed for each individual questionnare score due to high correlations across the different psychiatric questionnaires, whereas for the transdiagnostic analysis, we included all three psychiatric dimension scores in the same model, as correlation across variables was lessened in this formulation and thus more interpretable (only 3 moderately correlated variables *r* = 0.34–0.52, instead of 9 that ranged from *r* = 0.13–0.84). This allowed us to examine the association between CIT and various task measures, after the relationships with other factors (AD and SW) were controlled for.

### Action and confidence

A similar approach with mixed-effects models was used to analyse the basic relationship of questionnaire scores/psychiatric dimensions with *Action* or *Confidence* as dependent variables with the intercept as the random effect, controlled for age, gender and IQ.

### Computation model describing behaviour dynamics

To employ model-based analysis, we followed the same analysis steps as Vaghi *et al*.^13^. We calculated task prediction error (PE: distance between the particle landing location and the centre of the bucket) and human learning rate (LR^h^: change in chosen bucket position from *t* to *t* + 1 divided by PE on trial *t*) for each trial. Trials where LR^h^ exceeded the 99th percentile (LR^h^ > 7.75) and PE = 0 are thought to be unrelated to error-driven learning^12^, and were thus excluded from analyses with the model parameters (3.05% of total trials).

In the task, several evidence sources were available to participants (e.g. new information gained, surprise from unexpected outcomes and uncertainty of their belief) to estimate the mean of the generative distribution in order to position their bucket at where they hope to catch the greatest number of particles. We fitted a quasi-optimal Bayesian learning model, identical to the model specified in Vaghi *et al*.^13^ using functions freely available online from the original study, to particle landing location data in MATLAB R2018a (The MathWorks, Natick, MA). The model estimates variables thought to underlie task dynamics. This included *PE*^*b*^ (model prediction error, an index of recent outcomes), *CPP* (change-point probability, a measure representing the belief of a surprising outcome) and *RU* (relative uncertainty, the uncertainty owing to the imprecise estimation of the distribution mean; labelled as *(1*−*CPP)* * *(1*−*MC)* in Vaghi *et al*.). Similar to Vaghi *et al*., the correlations between model parameters were moderately correlated, with the largest correlation between PE^b^ and CPP: *r*_*s*_ = 0.68 (see Supplementary Table S1). This is because large deviations necessarily induce a CPP of near 1 and small deviations a CPP near 0.

The reduced quasi-optimal Bayesian learner, in accordance with prior literature^2,12,13,17^, uses a delta-rule to update its estimate of belief about the particle landing location distribution:

$${B}_{t+1}={B}_{t}+{\alpha }_{t}\times {\delta }_{t}$$

*B* is the new belief estimate on each trial *t*, which is equal to a point estimation of the mean of the Gaussian distribution where particle locations were sampled (i.e. 1 to 360). Its update is dependent on the learning rate $\alpha$ (LR^b^) and model prediction error $\delta$ (PE^b^). PE^b^ is calculated as the difference between the belief estimate *B*_*t*_ and the new particle landing location *X*_*t*_ and is a measure of information gained from the most recent trial.

$${\delta }_{t}={X}_{t}-{B}_{t}$$

As with common reinforcement learning models, LR^b^ determines how much new information (PE^b^) will update the belief estimate. However, LR^b^ is dynamic in the current model i.e. can change on every trial. If LR^b^ = 0, new evidence has had no impact on the update of the belief estimate, while LR^b^ = 1 suggests that the new belief estimate is entirely determined by the most recent outcome. The magnitude of LR^b^ is dependent the statistics of environment with the equation:

$${\alpha }_{t}={\Omega }_{t}+(1-{\Omega }_{t})(1-{\nu }_{t})$$

The first term, the change-point probability $\,\Omega$ (CPP), represents an estimate of how likely a change in particle location distribution mean has occurred on a given trial. The second term, model confidence $\nu$ (MC), represents the uncertainty due to an inaccurate estimation of the mean. For regression analyses, $(1-\Omega )\,(1-\nu )$ was labelled as RU (as the additive inverse of MC is relative uncertainty). These two components allow the model to appropriately update belief according to (i) unexpected changes in the environment (change-points) and (ii) the uncertainty about the distribution mean—thus informing when to disregard outliers, for example, when the mean is certain. New outcomes are more influential when the model believes that the distribution mean has changed (i.e. CPP is large) or is less sure about the true distribution mean (i.e. MC is small).

The model generates CPP as the relative likelihood that a new particle location is sampled from a new distribution i.e. during a change-point (mean determined by a uniform distribution *U* over all 360 possible locations), or drawn from the same Gaussian (*N*) where the current belief estimate *B*_*t*_ is centered upon. These are influenced by the hazard rate *H*, the probability that the mean of the distribution has changed. We set *H* equal to the hazard rates of the task trials (which were either H = 0.025 or 0.125, depending on the block condition). When the probability of the new particle location coming from a new distribution is high, CPP will be close to 1.

$${\Omega }_{t}=\frac{U({X}_{t}\,|\,1,360)H}{U({X}_{t}\,|1,360)H+N({X}_{t}\,|{B}_{t},{\sigma }_{t}^{2})(1-H)}$$

${\sigma }_{t}^{2}$ is the estimated variance of the predictive distribution, which consists of the variance of the generative Gaussian distribution ${\sigma }_{N}^{2}$ and the generative variance modulated by MC ($\nu$). As the predictive distribution variance is dependent on MC, it is larger than the generative variance where MC is the smallest (i.e. after change-points, where uncertainty of the new distribution mean is the highest) and will slowly reduce towards the generative variance. Thus, the model describes particle locations occurring after a change-point as less likely sampled from another new distribution.

$${\sigma }_{t}^{2}={\sigma }_{N}^{2}+\frac{(1-{\nu }_{t}){\sigma }_{N}^{2}}{{\nu }_{t}}$$

Lastly, MC is computed for the subsequent trial with a weighted average of the generative variance conditional on a change-point (first term), generative variance conditional on no change-point (second term), and variance due to the model’s uncertainty of whether a change-point occurred (third term) in the numerator. The denominator includes the same terms plus just the generative distribution variance (${\sigma }_{N}^{2}$) representing the uncertainty owing to noise. The full equation is as follows:

$${\nu }_{t+1}=\frac{{\Omega }_{t}{\sigma }_{N}^{2}+(1-{\Omega }_{t})(1-{\nu }_{t}){\sigma }_{N}^{2}+{\Omega }_{t}(1-{\Omega }_{t}){({\delta }_{t}{\nu }_{t})}^{2}}{{\Omega }_{t}{\sigma }_{N}^{2}+(1-{\Omega }_{t})(1-{\nu }_{t}){\sigma }_{N}^{2}+{\Omega }_{t}(1-{\Omega }_{t}){({\delta }_{t}{\nu }_{t})}^{2}+{\sigma }_{N}^{2}}$$

### Influence of parameters on action and confidence

Regressions were constructed as mixed-effect models with all of the model parameters (where *PE*^*b*^ is taken as its absolute) and a *Hit* categorical predictor (previous trial was a hit or miss) as within-subject regressors, controlled for age, IQ and gender. These regressions control for shared variance. For the regression on *Action*, following prior literature^2,12,13,17^, all predictors except *PE*^*b*^ were implemented as interaction terms with *PE*^*b*^. For *Confidence*, we used a similar regression model but without the interaction term with *PE*^*b*^ and with the predictors z-scored at participant level. We obtained similar regression estimates as Vaghi *et al*.^13^, suggesting that action/confidence was appropriately updated in accordance with changes in these parameters (Supplementary Table S2). To investigate the relationship of the questionnaire scores and psychiatric dimensions with the influence of these parameters on action/confidence, we included these scores as z-scored fixed effect predictors in the basic model above (individual models were examined for each questionnaire score, while for the transdiagnostic analysis, all three psychiatric dimension scores were included together).

There was some evidence of heteroskedasticity in the association between psychiatric variables and task parameters. White’s tests indicated that the model of RU on confidence with psychiatric dimensions was heteroskedastic (*p* = 0.04, but not the other parameters: *p* > 0.12). We therefore estimated heteroskedasticity-consistent standard errors for all coefficients reported by the *vcovHC* function from the *sandwich* package in R, detailed in the Supplement (Supplementary Table S3). The results do not diverge from those reported in the main paper.

For details of all regression equations, see Supplemental Methods.

## Results

### Action-confidence decoupling is linked to various psychiatric phenomena

**Figure 2:**
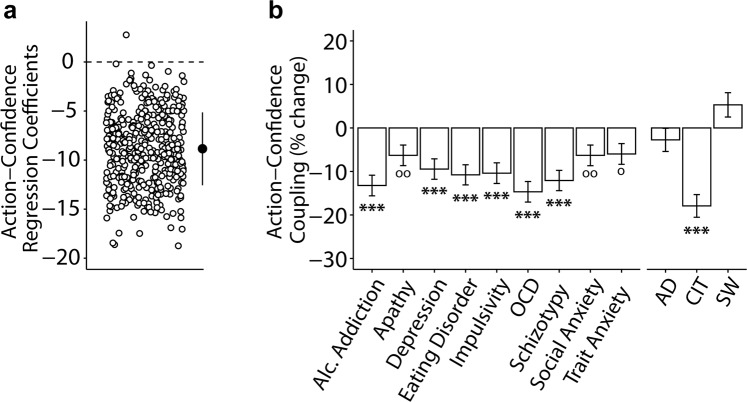
Action-confidence coupling and its relationship with questionnnaire scores/dimensions (controlled for IQ, age and gender). AD: anxious-depression, CIT: compulsive behaviour and intrusive thought, SW: social withdrawal. (**a**) Regression model where action update is predicted by confidence. Individual coefficients are represented by circles. Marker indicates the mean and standard deviation. As expected, regression coefficients were negative, such that higher confidence was associated with smaller updates to the bucket position (‘action’). (**b**) Associations between action-confidence coupling and questionnaire scores or psychiatric dimensions. All questionnaire scores predicted a decrease in action-confidence coupling. This decoupling relationship was strongest for the CIT dimension. Each questionnaire score was examined in a separate regression, while dimensions were included in the same regression model. The Y-axes shows the percentage change in the size of the action-confidence coupling effect as a function of 1 standard deviation increase of questionaire/dimension scores. Error bars denote standard errors. ^o^p < 0.05, ^oo^p < 0.01 uncorrected; *p < 0.05, **p < 0.01, ***p < 0.001 corrected. Results are Bonferroni corrected for multiple comparisons over number of psychiatric independent variables investigated. See also Supplementary Fig. S8.

In line with prior research, size of action updates (bucket position difference from trial *t* and *t* + 1) were strongly related to confidence within-subjects (*β* = −8.85, *Standard Error (SE)* = 0.31, 95% Confidence Interval (CI) [−9.45, −8.25], *p* < 0.001) (Fig. 2a), such that lower confidence was linked to larger updates, in the sample as a whole. Previous work by Vaghi *et al*. found that OCD patients exhibited reduced coupling between action and confidence compared to controls, which was correlated to the severity of self-reported OCD symptomology within the patient sample^13^. We tested the latter in a general population sample and replicated this result; OCD symptom severity was associated with significantly lower action-confidence coupling (*β* = 1.30, *SE* = 0.21, 95% CI [0.89, 1.71], *p* < 0.001, corrected) (Fig. 2b). However, we found that this relationship was profoundly non-specific—all nine questionnaire scores tested showed a similar pattern of reduced coupling. 6/9 questionnaires (alcohol addiction, depression, eating disorders, impulsivity, OCD and schizotypy) had significant decoupling at *p* < 0.001 corrected; the remaining three (apathy, social anxiety, trait anxiety) trended in the same direction, but did not survive Bonferroni correction for multiple comparisons.

### Transdiagnostic analysis shows a more specific pattern

When we refactored the data into three transdiagnostic dimensions defined previously in the literature, a profoundly different picture emerged. CIT (‘compulsive behaviours and intrusive thought’) was the only dimension to show decreased action-confidence coupling (*β* = 1.57, *SE* = 0.23, 95% CI [1.13, 2.01], *p* < 0.001, corrected) (Fig. 2b). Thus, while reductions in action-confidence coupling show broad and non-specific relationships to all questionnaire scores studied here, this pattern is explained by a single transdiagnostic dimension.

### Compulsivity is linked to inflated confidence, not aberrant action-updating

**Figure 3:**
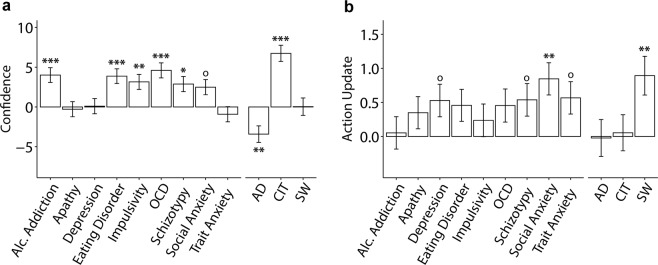
Associations between questionnaire scores, or dimensions (controlled for IQ, age and gender) with confidence or action. AD: anxious-depression, CIT: compulsive behavior and intrusive thought, SW: social withdrawal. (**a**) Associations with confidence rating on each trial. Most of the questionnaires scores were positively associated with confidence. However, refactoring into transdiagnostic dimensions revealed previously obscured bidirectional associations. AD was linked to decreased confidence, while CIT was linked to increased confidence. (**b**) Associations with action updates (i.e. bucket movement from one trial to the next). Only social anxiety was associated with an increased tendency to move the bucket, and this was similarly captured by, and specific to, the SW dimension. The Y-axes shows the percentage decrease in the size of the confidence or action update as a function of 1 standard deviation increase of questionnaire/dimension scores. Error bars denote standard errors. ^o^p < 0.05, ^oo^p < 0.01 uncorrected; *p < 0.05, **p < 0.01, ***p < 0.001 corrected. Results are Bonferroni corrected for multiple comparisons over the number of psychiatric independent variables investigated.

Prior work using this task in diagnosed patients found no confidence biases in OCD, but abnormalities in action-updating. Using our transdiagnostic method, we found a different pattern of results. CIT was associated with higher overall confidence levels (*β* = 6.74, *SE* = 1.02, 95% CI [4.75, 8.73], *p* < 0.001, corrected), and not changes in action-updating. In line with prior work, we found that AD (‘anxious-depression’) was associated with lower confidence (*β* = −3.42, *SE* = 1.04, 95% CI [−5.45, −1.39], *p* = 0.003, corrected) (Fig. 3a). Because OCD patients tend to have high levels of AD, this finding suggests that a transdiagnostic method may be necessary to reveal the role confidence plays in clinical phenotypes, which could otherwise be obscured within the heterogeneous diagnostic category. In terms of action-updating, only SW (‘social withdrawal’) showed an association, such that participants scoring high in this dimension tended to move the bucket more (*β* = 0.89, *SE* = 0.28, 95% CI [0.34, 1.45], *p* = 0.005, corrected) (Fig. 3b).

### Confidence in compulsivity is less sensitive to unexpected outcomes, environment uncertainty and positive feedback

The previous analyses suggest that confidence in compulsive individuals is both inflated and decoupled to behaviour. To understand the mechanism behind this, we tested the extent to which confidence estimates were sensitive to multiple factors that should drive belief-updating. Specifically, prior work has shown that trial-wise adjustments in behaviour are influenced by 1) information gained from the most recent change in particle location (PE^b^; model prediction error), 2) surprising large particle location changes owing to change-points (CPP; change-point probability) and 3) uncertainty of one’s belief about the particle landing location distribution mean (RU; relative uncertainty)^17^. To separate the contributions of these factors, we computed the three normative parameters with a quasi-optimal Bayesian model^2,12,13,17^ (see Methods) to the sequence of particle locations experienced by each participant.

**Figure 4:**
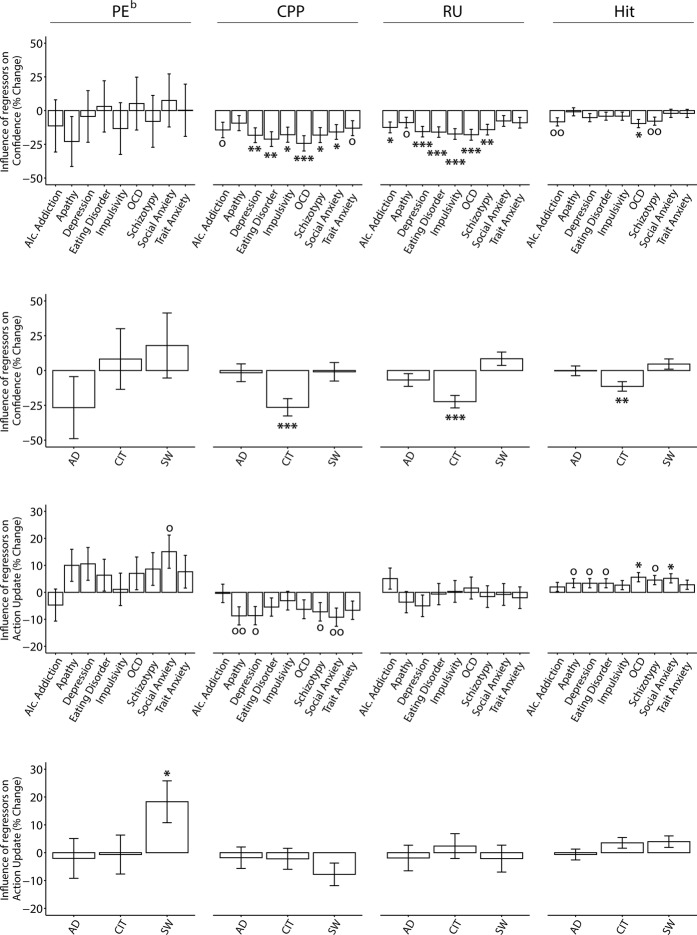
Regression analyses of trial-wise confidence and action adjustments with questionnaire scores/dimensions, controlled for age, IQ and gender. Predictors for confidence and action update regressions include model parameters PE^b^ (model prediction error), CPP (change point probability), RU (relative uncertainty) and Hit (previous trial was a hit). They are indicated at the top of the figure for each column inset. Each questionnaire score was examined in a separate regression, whereas dimensions were included in the same model (AD: anxious-depression, CIT: compulsive behavior and intrusive thought, SW: social withdrawal). Error bars denote standard errors. The Y-axes indicate the percentage change in the size of the model parameter on confidence or action update effect as a function of 1 standard deviation of questionnaire/dimension scores. ^o^p < 0.05, ^oo^p < 0.01 uncorrected; *p < 0.05, ***p < 0.001 corrected. Results are Bonferroni corrected for multiple comparisons over the number of psychiatric independent variables investigated. See also Supplementary Table S3 and Fig. S5.

We analysed trial-wise confidence using regression models with these parameters including a categorial Hit regressor (previous trial was a hit or miss), and controlling for age, gender and IQ. Overall, confidence was influenced by PE^b^, CPP, RU and Hit (Supplementary Table S2). The CIT symptom dimension was associated with a significantly diminished influence of CPP (*β* = 0.05, *SE* = 0.01, 95% CI [0.03, 0.08], *p* < 0.001, corrected), RU (*β* = 0.05, *SE* = 0.01, 95% CI [0.03, 0.07], *p* < 0.001, corrected) and Hit (*β* = −0.03, *SE* = 0.01, 95% CI [−0.05, −0.01], *p* = 0.003, corrected) on confidence (Fig. 4 and Supplementary Fig. S5a). In other words, confidence estimates in CIT were less sensitive to unexpected outcomes, the uncertainty of the true distribution mean and whether the previous particle was caught (i.e. correct trial). These results suggest that confidence in highly compulsive individuals is not only inflated, it is also disconnected to several sources of environmental evidence. Interestingly, the failures in utilizing evidence do not explain away overall inflated confidence observed in CIT (*β* = 6.78, *SE* = 1.02, 95% CI [4.79, 8.76], p < 0.001, corrected), suggesting these are at least partially distinct metacognitive failures. There were no associations between AD or SW and the extent to which evidence influenced confidence (Fig. 4).

### Action updates in compulsivity respond appropriately to evidence

Using the same analysis approach described for confidence, we found that trial-wise action adjustments were influenced by all model parameters (Supplementary Table S2). However, in contrast to confidence, CIT was not linked to changes in the influence of any of the parameters on action (Fig. 4 and Supplementary Fig. S5b). SW was related to a significant increased influence of PE^b^, suggesting that individuals high in this trait had an increased tendency to update their action with every new outcome (*β* = 0.06, *SE* = 0.02, 95% CI [0.02, 0.09], *p* < 0.05, corrected) (Fig. 4). There were no associations with AD. Additional analyses in the Supplement show that when demographics are not controlled for, some modest associations between action-updating and compulsivity emerge that correspond to those reported previously in OCD^13^ (Supplementary Fig. S7).

## Discussion

In this study, we demonstrated that a breakdown in the relationship between explicit belief (confidence) and behaviour is associated with a transdiagnostic psychiatric dimension—compulsive behaviour and intrusive thought (CIT). This decoupling arises from abnormalities in belief, rather than behaviour. Individuals high in CIT exhibited overall inflated confidence estimates and failures in utilizing unexpected outcomes, belief uncertainty and positive feedback to inform their confidence levels appropriately. In contrast, action-updating in response to these factors did not differ as a function of severity of CIT. Our findings suggest a dysfunctional metacognitive mechanism in compulsivity that implicates difficulty in updating the explicit model of the world in response to various sources of evidence.

Existing models of compulsivity propose that deficits in goal-directed control leave individuals vulnerable to establishing compulsive habits^28^. Supporting evidence primarily come from behavioural tests, where OCD patients (and other compulsive disorders) have difficulty exerting control over well-trained habits when motivations change (i.e. a devaluation test)^5,6,9,29^. Other tasks have shown that compulsive patients have deficits utilizing a world model to make choices prospectively (even when habits are not present), relying solely on reinforcement (i.e. feedback)^8,10^. Our current finding, that high compulsive individuals fail to update their world-model in response to several types of evidence, is an important extension of this literature. The challenge facing compulsive individuals has until now been presumed to be the implementation of the model rather than its generation and/or maintenance^28^. This implicates not just our understanding of compulsive disorders, but also their treatment. Recent work has shown metacognitive skills can be improved though adaptive training^30^; there may be scope for trialing these treatments for psychiatric populations where compulsivity is an issue.

Confidence was not just unresponsive to various sources of evidence, it was also inflated in compulsive individuals. This finding replicates prior work using this same transdiagnostic methodology that examined confidence in the context of perceptual decision-making^15^, showing elevated confidence in compulsivity extends to reinforcement learning—which is of course highly relevant for the behavioural aspects of compulsivity. However, this finding of increased confidence in compulsivity may seem at odds with prior research that overall suggests a decrease of confidence in OCD^31^. Critically, these studies have primarily been conducted with patient versus control comparisons and with respect to confidence in memory, rather than decision-making. There are several factors that are important to consider as this field progresses. Firstly, many memory tasks have not controlled for differences in performance in the way that tasks in the perceptual/reinforcement learning domains have been able to, which introduces a confound to interpretation^32^. If subjects perform worse at the memory task, untrue conclusions about under-confidence can arise. This is the case in some, but not all, tasks that have studied confidence in memory in OCD. Secondly, it has been demonstrated that metacognition is not a unitary phenomenon; there is specialisation in distinct brain regions for confidence in perceptual versus memory domains^33^. We therefore might reasonably expect different patterns of dysfunction in OCD, depending on the domain under study. Finally, and perhaps most importantly, these prior studies are based on group comparisons (usually patient versus control) and cannot account for the influence of comorbid symptomatology (e.g. depression and anxiety) on confidence. Given depression is associated with decreases in confidence, the confluence of these symptoms might serve to mask the true direction of the relationship between compulsivity and confidence.

In instances when confidence diverges from action, prior work has suggested confidence estimates may be corrupted by noise, internal states or a continued/lack of evidence processing^34,35^. Coupled with the finding that confidence is less informed by several sources of evidence in high compulsive individuals, it is possible that inflated confidence in compulsivity observed here arose through some unmodeled form of information processing. In constrast, we found that actions were updated normally in response to feedback in high compulsives, which accords with prior work showing that basic reinforcement learning in compulsive patient groups (i.e. ‘model-free’ learning) is intact^10,9^. That said, a previous study using this task found increased action-updating tendencies in OCD^13^. Here, the discrepancy does not appear to be explained by the superiority of a transdiagnostic approach per se, but perhaps our ability to control for some demographic confounds (see Supplementary Fig. S7). Instead, we found that social withdrawal (SW) was associated with a higher sensitivity to new information affecting action. Though this result was not hypothesized, it aligns with prior research suggesting that socially anxious people engage in greater performance monitoring^36^ and have higher sensitivity to learning from feedback^37^.

Beyond the specific results of this study with respect to confidence and compulsivity, our data highlight the benefit of transdiagnostic dimensions over traditional modes of phenotyping. When we examined questionnaires that are ubiquitous but rarely compared to one another in clinical research, we found strikingly non-specific patterns of association with task variables. For example, all nine questionnaires showed an association with action-confidence coupling in the same direction (6/9 surviving strict correction). In contrast, the compulsive factor was the only transdiagnostic dimension to show an association. In addition to resolving issues with collinearity across questionnaires, this approach also resolves issues associated with the heterogeneity within them. For example, severity of neither depression nor anxiety was associated with decreases in confidence using a standard clinical questionnaire, but the anxious-depression (AD) dimension was. In comparison to work with diagnosed patients, the benefits of the transdiagnostic approach are the same. Prior work using this task found no difference in OCD patients’ mean confidence ratings compared to healthy controls^13^, while we found a strong a reproducible association between CIT and inflated confidence and AD and diminished confidence^15^, at least in the context of decision-making. Given that OCD is frequently co-morbid with anxiety disorders (over 75%^38^), which has an opposing relationship to confidence, it is no surprise that differences between OCD patients and controls are obscured when transdiagnostic dimensions are not considered. Together, these data suggest that transdiagnostic phenotyping may, at least in some domains, provide a closer fit to underlying brain processes than DSM distinctions.

This study was not without limitation. Our study was conducted online, thus experimenter control of the testing environment was virtually non-existent. Prior studies have however shown that cognitive data collect online, albeit noisier, is valid^39^. Similarly, self-report psychiatric scores are on-par with the general population^40^ and relationships between cognition and clinical measures are mirrored across testing modalities^11,41^. Additionally, as the task was adapted for web-based testing, response navigation was controlled by keyboard presses (right and left response keys to direct clockwise and counter-clockwise rotations) and not a rotor controller as in Vaghi *et al*.^13^, which could plausibly feel less natural and thus increase noise in spatial update measure. However, we were able to reproduce basic main effects of model parameters on action from Vaghi *et al*., suggesting that the different response modality did not affect our ability to study action updating behaviour. We observed similar basic main effects of model parameters on action updating as in the original paper (Supplementary Table S2). With respect to the psychiatric dimensions, we not only reproduced the factor structure from a prior paper with our current data (Supplementary Fig. S6), we used the factor weights from this prior publication^11^ to transform raw questionnaire scores into transdiagnostic factors for analysis. This ensured independence and underscores the robustness and reproducibility of these factors and their association to cognition.

The extent to which these results are applicable to diagnosed patients is not something we can directly address here. However, it is notable that we replicated the association between OCD symptoms and action-confidence decoupling observed in a clinical sample that were tested in-person^13^. The same applies to goal-directed planning, which is both deficient in patients tested in-person^9^ and correlated with OCD symptoms in the general population tested in-person^41^ and online^11^. Notably, recent work in a mixed generalised anxiety disorder and OCD patient sample that were tested online found that goal-directed deficits were more strongly associated with the compulsivity dimension than OCD diagnosis status, underscoring the importance of transdiagnostic methods for delineating specific associations between cognition and clinical phenomenology that can be masked when examining diagnostic status alone^42^. Future research is needed to investigate if the association between inflated confidence and compulsivity is similarly evident in diagnosed patients, tested in-person. More concerted, multi-centre, efforts are required to achieve the large samples necessary to undertake this work, if it is to take place in-person rather than online.

To conclude, we highlighted how a transdiagnostic methodology can be crucial for uncovering specific associations between pathophysiology and clinical symptoms. This method has several strengths: it directly addresses the issue of psychiatric co-morbidity, helps us to achieve higher statistical power and thus promotes reproducibility, and makes research faster, more efficient and even more representative^43^. The definition of compulsivity employed here was generated in an independent study and is not intended to be fixed or final. Rather, its application to this independent dataset is intended to show the general potential that transdiagnostic approaches have for dealing with the issues of comorbidity and individual differences faced both in research and practice in psychiatry. We used this method to show that compulsive behaviour and intrusive thought is associated with reduced action-confidence coupling, inflated confidence and diminished influence of evidence on confidence estimates. Our findings suggest that compulsivity is linked to problems in developing an explicit and accurate model of the decision space, and this might contribute to broader class of problems these individuals face with goal-directed planning and execution.

## Supplementary information

Supplementary Info.

## Data Availability

The code and data to reproduce the main analyses are freely available in an Open Science Framework (OSF) repository, at https://osf.io/2z6tw/.
