# Author Correction: Transdiagnostic Phenotyping Reveals a Host of Metacognitive Deficits Implicated in Compulsivity

**DOI:** 10.1038/s41598-021-95184-3

**Published:** 2021-07-27

**Authors:** Tricia X. F. Seow, Claire M. Gillan

**Affiliations:** 1 School of Psychology, Trinity College Dublin, Dublin 2, Ireland; 2 Trinity College Institute of Neuroscience, Trinity College Dublin, Dublin 2, Ireland; 3 Global Brain Health Institute, Trinity College Dublin, Dublin, Ireland

Correction to: *Scientific Reports* https://doi.org/10.1038/s41598-020-59646-4, published online 19 February 2020

The Methods section in the original version of this Article was incomplete. The subsection ‘Self-report psychiatric questionnaires & IQ’ now reads:

“Participants completed a range of self-report psychiatric assessments after the behavioural task. To enable application of the transdiagnostic analysis with psychiatric dimensions described in previous studies^11,15^, we administered the same nine questionnaires assessing: *Alcohol addiction* using the Alcohol Use Disorder Identification Test (AUDIT)^18^, *Apathy* using the Apathy Evaluation Scale (AES)^19^, *Depression* using the Self-Rating Depression Scale (SDS)^20^, *Eating disorders* using the Eating Attitudes Test (EAT-26)^21^, *Impulsivity* using the Barratt Impulsivity Scale (BIS-10)^22^, *Obsessive-compulsive disorder* (OCD) using the Obsessive-Compulsive Inventory – Revised (OCI-R)^23^, *Trait anxiety* using the trait portion of the State-Trait Anxiety Inventory (STAI)^24^, *Schizotypy* scores using the Short Scales for Measuring Schizotypy^25^, and *Social anxiety* using the Liebowitz Social Anxiety Scale (LSAS)^26^. Consistent with the original study that defined these transdiagnostic traits^11^, item 13 on the Self-Rated Depression Scale^20^ was erroneously administered as “I am restless and can’t sleep”, instead of “I am restless and can’t keep still”. The order of these self-report assessments administered was fully randomized. Following the questionnaires, participants completed a Computerized Adaptive Task (CAT) based on items similar to that of Raven’s Standard Progressive Matrices (SPM)^27^ to approximate Intelligence Quotient (IQ).”

The original Article has been corrected.

