# Supplementary material for: Transdiagnostic Phenotyping Reveals a Host of Metacognitive Deficits Implicated in Compulsivity
Source: Sci Rep. 2020 Feb 19;10:2883. doi: 10.1038/s41598-020-59646-4 (PMC7031252; doi:10.1038/s41598-020-59646-4)
Supplement: Supplementary file 1 — Supplementary Info. [file 41598_2020_59646_MOESM1_ESM.pdf]

Supplemental Information
for
Transdiagnostic phenotyping reveals a host of metacognitive deficits implicated in compulsivity

Supplemental Methods

Exclusion criteria. Participants were excluded if they failed any of the following: (i) In the behavioural task, the confidence scale indicator would always start at either 25 or 75 on every trial. Participants who left their confidence rating as the default score for more than 60% of the trials (n > 180 trials) were excluded (N = 42). (ii) The task was also reset from the beginning if confidence ratings were left as the default score for >70% of the first 50 trials (56 participants (9.82%) restarted the task at least once). Those who had their task reset >5 times were excluded (N = 6). (iii) Participants who had more than 50% correlation between the default score and their selected confidence rating were excluded (N = 109). (iv) Participants with a lower mean confidence where the previous trial was correct than incorrect were excluded (N = 66). (v) Participants who incorrectly responded to a "catch" question within the questionnaires: "If you are paying attention to these questions, please select 'A little' as your answer" were excluded (N = 16).

Medication status. Participants were asked if they were currently taking medication for a mental health issue, and if so, to indicate the name, dosage and duration. 41 (9.38%) participants were currently medicated.

Action-confidence coupling. First, we measured the coupling between action updates (i.e. the tendency to move the bucket) and confidence. *Action* (the absolute difference of bucket position on trial t and t+1) was the dependent variable and *Confidence* (confidence level on

trial *t*+1) was the independent variable in a trial-by-trial regression analysis with age, gender and IQ as fixed effects co-variates (as with all subsequent analyses). Within-subject factors (the intercept and main effect of *Confidence*) were taken as random effects (i.e., allowed to vary across subjects). *Confidence* was z-scored within-participant, while the fixed effect predictors were z-scored across participant. If action and confidence are appropriately coupled, participants should move the bucket more (larger *Action*) when their confidence levels were low, producing a significant negative main effect of *Confidence* on *Action*. In the syntax of the *lmer* function, the regression was: Action ~ Confidence * (Age + IQ + Gender) + (1 + Confidence | Subject).

We then tested if psychiatric symptom severity was associated to changes in action-confidence coupling by including the total score for each questionnaire (*QuestionnaireScore*, z-scored) as a between-subjects predictor in the model above. Separate regressions were performed for each individual symptom due to high correlations across the different psychiatric questionnaires. The extent to which questionnaire total scores contribute to changes in action-confidence coupling is indicated by the presence of a significant *Confidence***QuestionnaireScore* interaction. A positive interaction effect indicates decreased action-confidence coupling (i.e., decoupling), while a negative interaction effect indicates greater action-confidence coupling. The model was specified as: Action ~ Confidence * (*QuestionnaireScore* + Age + IQ + Gender) + (1 + Confidence | Subject). For the transdiagnostic analysis, we included all three dimensions in the same model, as correlation across variables was lessened in this formulation and thus more interpretable (only 3 moderately correlated variables r = 0.34 - 0.52, instead of 9 that ranged from r = 0.13 - 0.84). We replaced *QuestionnaireScore* in the model formula described previously with three psychiatric dimensions (*AD, CIT, SW*) entered as z-scored fixed effect

predictors. The model was: Action ~ Confidence * (AD + CIT + SW + Age + IQ + Gender) + (1 + Confidence | Subject).

Action and confidence. To analyse the basic relationship between task-related variables and psychiatric dimensions, the analysis approach was the same, but simpler. Dependent variables were: 1) Size of bucket updates (*Action*) and 2) reported confidence (*Confidence*). The models were simply: Task Variable ~ AD + CIT + SW + Age + IQ + Gender + (1 | Subject).

Computation model describing behaviour dynamics. In the behavioural task, participants were required to learn the mean of the underlying generative distribution in order to position their bucket at where they hope to catch the greatest number of particles. Their belief on where the particle landing distribution mean could be guided by 1) information gained from the most recent outcome (i.e. moving the bucket with every small shift in particle location), 2) surprising large changes signalling a change in mean distribution (i.e. change-points) and 3) their uncertainty of the distribution mean based on particle landing location experience over trials. To separate these contributions, a quasi-optimal Bayesian computational learning model was used to estimate these parameters thought to underlie task dynamics with MATLAB R2018a (The MathWorks, Natick, MA) using functions from Vaghi *et al.*[1]. This included PE^b (model prediction error, an index of recent outcomes), *CPP* (probability that a trial was a change-point, a measure representing the belief of a surprising outcome) and *RU* (relative uncertainty, the uncertainty owing to the imprecise estimation of the distribution mean; labelled as *(1-CPP)*(1-MC)* in Vaghi *et al.* (Vaghi *et al.*, 2017)). These parameters (where PE^b is taken as its absolute) together with a *Hit* categorical predictor (previous trial was a hit or miss) were used to regress participant adjustments against the benchmark Bayesian model to investigate participant adjustments in reported confidence (*Confidence*; z-scored confidence level on trial *t*) and

bucket movements (*Action*) according to the particle landing locations experienced.

Influence of parameters on action and confidence. For the regression on *Action*, following Vaghi *et al.*[1] and prior literature[2–4], all predictors except PE^b were implemented as interaction terms with PE^b. For Confidence, we used a similar regression model but without the interaction term with PE^b and with the regressand and predictors z-scored at participant level. Regressions were constructed as mixed-effect models controlled for age, IQ and gender, with the interaction term and main effect of regressors as random effects. The model syntax was written as: Dependent Variable ~ (PE^b + CPP + RU + Hit)*(Age + IQ + Gender) + (1 + PE^b + CPP + RU + Hit | Subject).

To include psychiatric symptom severity in the same analysis model, we entered each psychiatric questionnaire score as an additional z-scored fixed effect predictor into the basic model above, where the equation was: Dependent Variable ~ (PE^b + CPP + RU + Hit)*(*QuestionaireScor*e + Age + IQ + Gender) + (1 + PE^b + CPP + RU + Hit | Subject). For confidence, a positive interaction between a symptom score and PE^b, *CPP, RU* indicates that higher scores on that symptom are associated with a decrease in influence of these parameters on confidence. The converse was applicable for significant *Hit*QuestionaireScore* interactions (as main effect of *Hit* on *Confidence* is opposite signed). For action, as main effect of the parameters on *Action* is inverse from the main effects on *Confidence*, significant parameter**QuestionaireScore* interactions are interpreted in reverse. For the transdiagnostic analysis, we included all three dimensions in the same model by replacing *QuestionnaireScore* with three psychiatric dimensions (*AD, CIT, SW*) entered as z-scored fixed effect predictors. The model was: Dependent Variable ~ (PE^b + CPP + RU + Hit)*(AD + CIT + SW + Age + IQ + Gender) + (1 + PE^b + CPP + RU + Hit | Subject).

For visualization purposes, the main effects of the four predictors were correlated with CIT severity, where Spearman's correlation was used to measure the association between symptom dimension severity and the influence of the learning parameters on action update/confidence (Figure S5).

Influence of metacognitive parameters on action-confidence coupling in compulsivity. We investigated how confidence bias and participants' sensitivity to feedback on confidence were related to action-confidence coupling. We obtained individual beta coefficients from the basic regression model of the model parameters (*PE^b, CPP, RU and Hit*) on confidence from the mixed model equation: Confidence ~ (PE^b + CPP + RU + Hit)*(AD + CIT + SW + Age + IQ + Gender) + (1 + PE^b + CPP + RU + Hit | Subject), individual beta coefficients regression of action on confidence from the equation: Action ~ Confidence * (Age + IQ + Gender) + (1 + Confidence | Subject) and participants' mean confidence level. We regressed each subjects' coefficients for the effect of model parameters on confidence and their mean confidence level against action-confidence in a linear regression, with all regressors taken as z-scored fixed effect predictors. The equation was: Action on Confidence ~ PE^b on Confidence + CPP on Confidence + RU on Confidence + Hit on Confidence + Mean Confidence. To specifically examine how these factors were related to action-confidence coupling in compulsivity, we compared the main effect of CIT on action-confidence coupling in a model with above metacognitive factors: Action on Confidence ~ PE^b on Confidence + CPP on Confidence + RU on Confidence + Hit on Confidence + Mean Confidence + AD + CIT + SW and without the above metacognitive factors: Action on Confidence ~ AD + CIT + SW. Heteroskedasticity-consistent standard errors for all coefficients are reported by the *vcovHC* function from the *sandwich* package in R.

As expected, action-confidence coupling was significantly related to PE on confidence: β = 1.91, *SE* = 0.18, $p < 0.001$, CPP on confidence: β = 4.50, *SE* = 0.40, $p < 0.001$, RU on confidence: β = -1.21, *SE* = 0.37, $p = 0.001$, Hit on confidence: β = -1.53, *SE* = 0.14, $p < 0.001$) and marginally to confidence bias (β = -0.13, *SE* = 0.07, p = 0.07). When we included compulsivity in the model above, we found that the original effect of compulsivity on action-confidence coupling was reduced but remained significant (CIT: β = 0.32, *SE* = 0.09, p = 0.002, corrected), suggesting that decreased action-confidence coupling is only partially explained by the multiple metacognitive parameters of the task.

Supplemental Figures and Tables

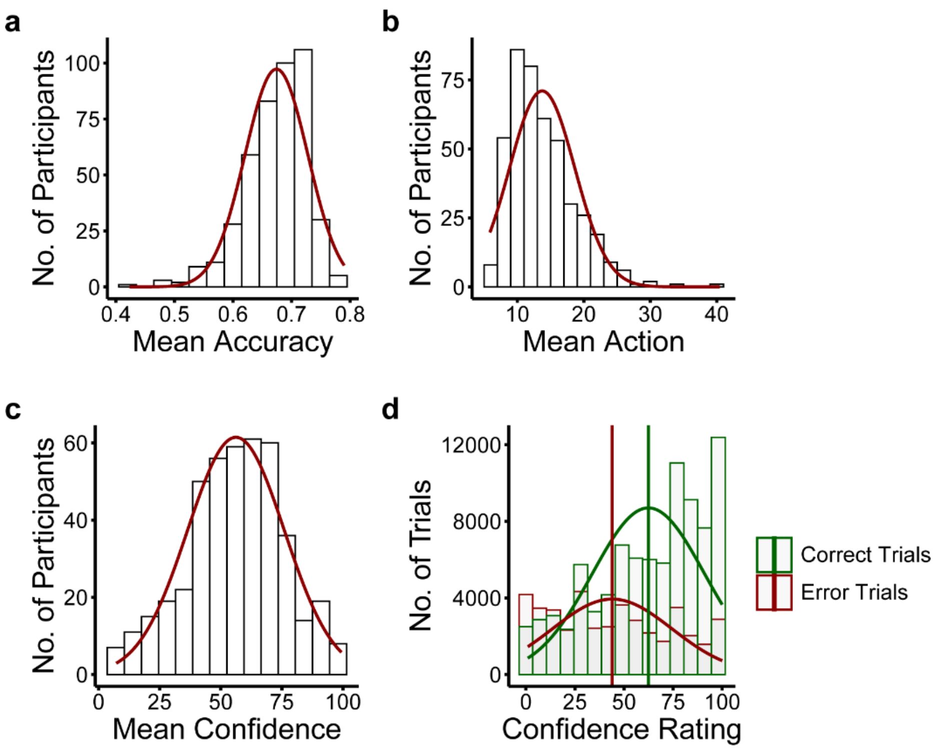

Supplementary Figure S1. Behavioural results. Across participants, the distribution of:
(a) Mean accuracy.
(b) Mean action (the tendency to move the bucket).
(c) Mean confidence level.
(d) Confidence ratings for correct (green) and incorrect (red) trials. Vertical lines denote mean confidence level for respective trial type.

Across participants, mean accuracy ranged from 42.33% to 79.00% (mean = 67.42%, SD = 5.38%; Figure S2a), mean action (tendency to move bucket position) ranged from 5.88 to 40.44 (mean = 13.74, SD = 4.91, Figure S2b) and mean confidence level ranged from 7.21 to 99.39 across participants (mean = 56.19, SD = 19.85; Figure S2c). Performance accuracy accounted for only 1.7% of the variance in confidence levels (between-subject correlation: $r = 0.13$, $p < 0.009$). Participants were using the confidence scale appropriately, giving higher confidence after correct trials (mean = 62.42, SD = 28.53), and lower confidence after incorrect trials (mean = 43.98, SD = 30.45) (Figure S2d).

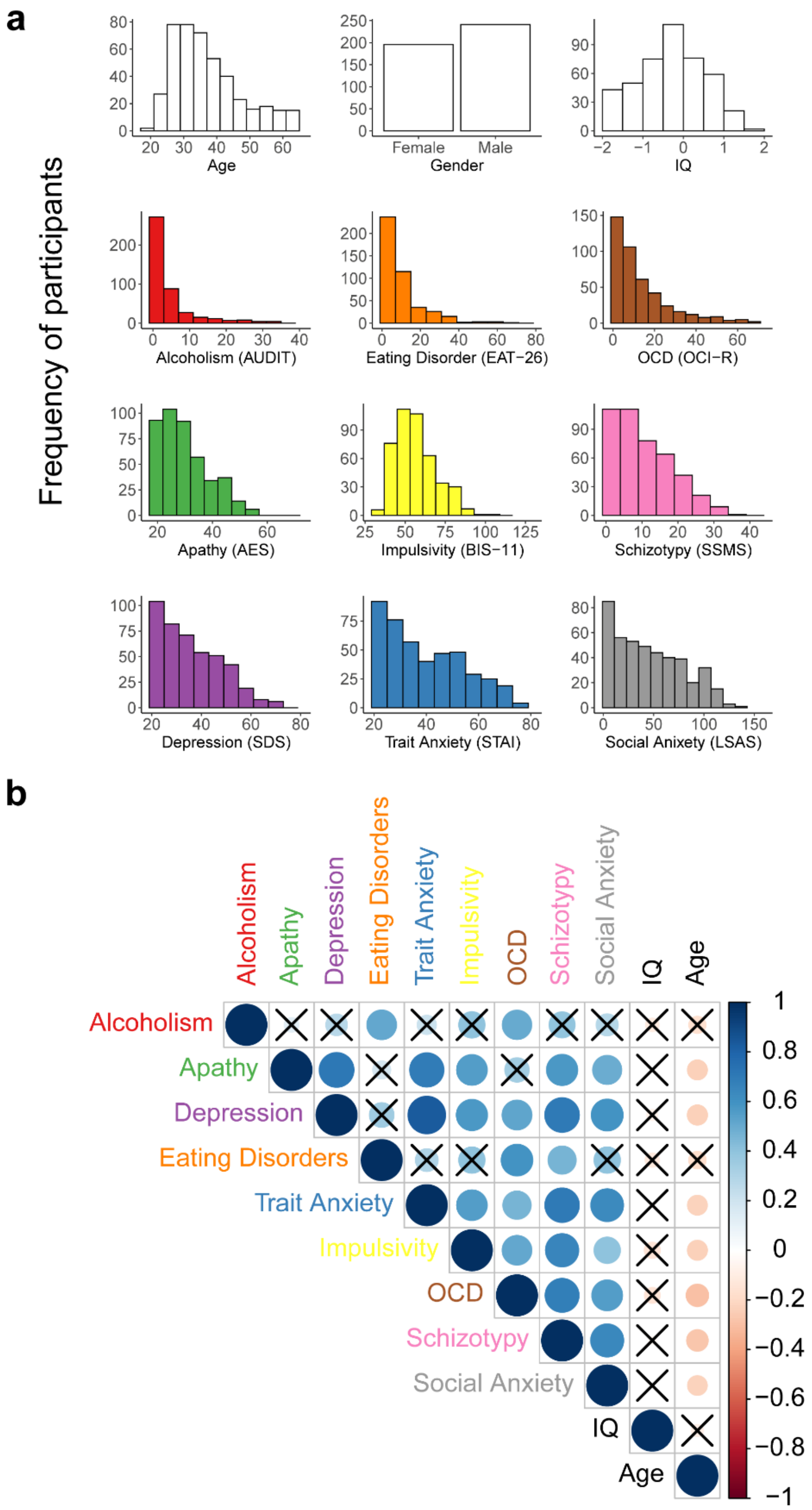

Supplementary Figure S2. Demographics and self-reported psychopathology spread.
(a) Age, IQ and questionnaire score distributions across participants.
(b) Correlation matrix of mean scores of the nine questionnaires, age and IQ. Colour scale indicates correlation coefficient, size of colour patch indicates significance. X denotes correlation fails 95% Confidence Interval.

Supplementary Table S1. Spearman's correlation between Bayesian Model Parameters (and Hit).

	**PE^b**	**CPP**	**RU**	**Hit**
**PE^b**	*1*			
**CPP**	*0.68*	*1*		
**RU**	*0.09*	*0.46*	*1*	
**Hit**	*-0.55*	*-0.44*	*-0.12*	*1*

Supplementary Table S2. Effects of Bayesian Model Parameters on Action and Confidence. SE = standard Error, CI = confidence interval.

Predictor	***β (SE)***	***95% CI***	***t-value***	***p-value***
	Regression on Action			
PE^b	*0.33 (0.02)*	*[0.27, 0.38]*	*11.61*	*< 0.001 ****
CPP	*0.46 (0.02)*	*[0.41, 0.50]*	*20.06*	*< 0.001 ****
RU	*1.37 (0.08)*	*[1.21, 1.52]*	*17.25*	*< 0.001 ****
Hit	*-0.77 (0.02)*	*[-0.81, -0.73]*	*-40.54*	*< 0.001 ****
	Regression on Confidence			
PE^b	*-0.04 (0.01)*	*[-0.06, -0.02]*	*-3.57*	*< 0.001 ****
CPP	*-0.20 (0.02)*	*[-0.24, -0.17]*	*-12.16*	*< 0.001 ****
RU	*-0.24 (0.01)*	*[-0.27, -0.21]*	*-17.15*	*< 0.001 ****
Hit	*0.26 (0.01)*	*[0.23, 0.29]*	*22.84*	*< 0.001 ****

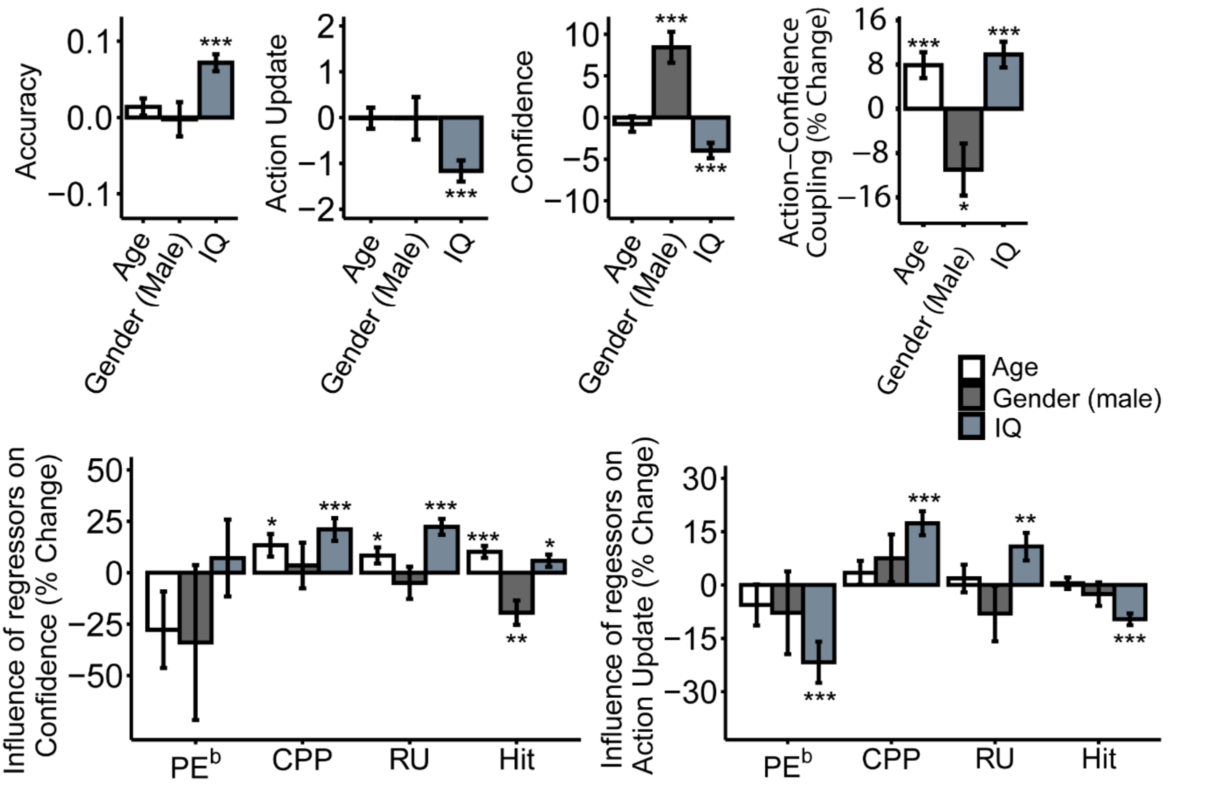

Supplementary Figure S3. Associations between age, gender and IQ with accuracy, action update, reported confidence, action-confidence coupling or the influence of the model predictors (PE^b, CPP, RU) and Hit on confidence/action update. Error bars denote standard errors. The Y-axes indicates the change/percentage change in each dependent variable as a function of 1 standard deviation increase of demographic scores. *$p < 0.05$, **$p < 0.01$, ***$p < 0.001$.

We tested in an exploratory fashion for relationships of task accuracy, action and confidence with age, IQ and gender (Figure S4). IQ was found to predict better performance ($\beta = 0.07$, $SE = 0.01$, 95% CI [0.05, 0.09], p < 0.001), lower action updating, ($\beta = -1.16$, $SE = 0.23$, 95% CI [-1.62, -0.71], $p < 0.001$) and lower confidence ($\beta = -3.97$, $SE = 0.92$, 95% CI [-5.77, -2.17], $p < 0.001$). Additionally, gender (male) was associated with higher confidence ($\beta = 8.43$, $SE = 1.85$, 95% CI [4.81, 12.06], $p < 0.001$).

IQ, age and gender were controlled for in all analyses. Increased action-confidence coupling was associated to age ($\beta = -0.70$, $SE = 0.21$, 95% CI [-1.10, -0.29], $p < 0.001$), and IQ ($\beta = -0.87$, $SE = 0.21$, 95% CI [-1.27, -0.46], $p < 0.001$) while decreased in males ($\beta = 0.97$, $SE =$

0.42, 95% CI [0.16, 1.78], $p = 0.02$). For the model-based trial-wise analyses, age was related to an increased influence of CPP ($\beta = -0.03$, $SE = 0.01$, 95% CI [-0.05, -0.01], $p = 0.02$), RU ($\beta = -0.02$, $SE = 0.01$, 95% CI [-0.04, -0.002], $p = 0.03$) and Hit ($\beta = 0.02$, $SE = 0.01$, 95% CI [0.01, 0.04], $p = 0.03$) on confidence. Males were associated to an increased influence of Hit ($\beta = -0.05$, $SE = 0.02$, 95% CI [-0.08, -0.02], $p = 0.001$) on confidence, while IQ predicted increased influence of CPP ($\beta = -0.05$, $SE = 0.01$, 95% CI [-0.06, -0.02], $p < 0.001$), RU ($\beta = -0.05$, $SE = 0.01$, 95% CI [-0.07, -0.03], $p < 0.001$) and Hit ($\beta = 0.02$, $SE = 0.01$, 95% CI [0.0003, 0.03], $p = 0.05$) on confidence. For action update, only IQ effects were significant – it was related to an increase in CPP ($\beta = 0.08$, $SE = 0.02$, 95% CI [0.05, 0.11], $p < 0.001$) and RU ($\beta = 0.15$, $SE = 0.05$, 95% CI [0.04, 0.25], $p = 0.006$) influence, and decreased PE[b] ($\beta = -0.07$, $SE = 0.02$, 95% CI [-0.11, -0.03], $p < 0.001$) and Hit ($\beta = 0.07$, $SE = 0.01$, 95% CI [0.05, 0.10], $p < 0.001$) influence on action update.

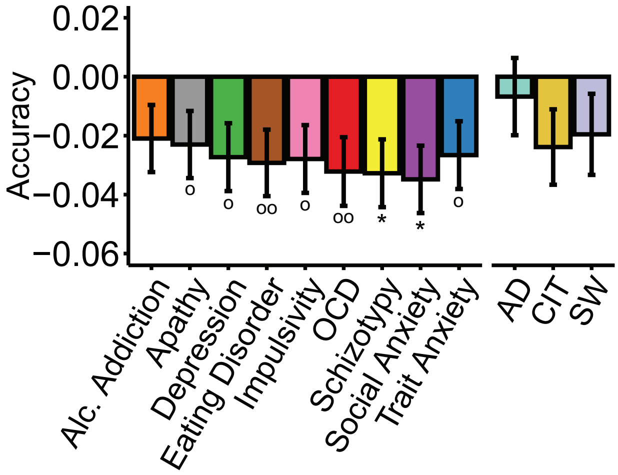

Supplementary Figure S4. Associations between accuracy (hit (1) or miss (0)) with questionnaire scores or transdiagnostic dimensions, controlled for age, IQ and gender. Error bars denote standard errors. The Y-axis indicates the change in accuracy as a function of 1 standard deviation of questionnaire/dimension scores. $^{o}p < 0.05$, $^{oo}p < 0.01$ uncorrected, $^{*}p < 0.05$. Results are Bonferroni corrected for multiple comparisons over number of questionnaires/dimensions.

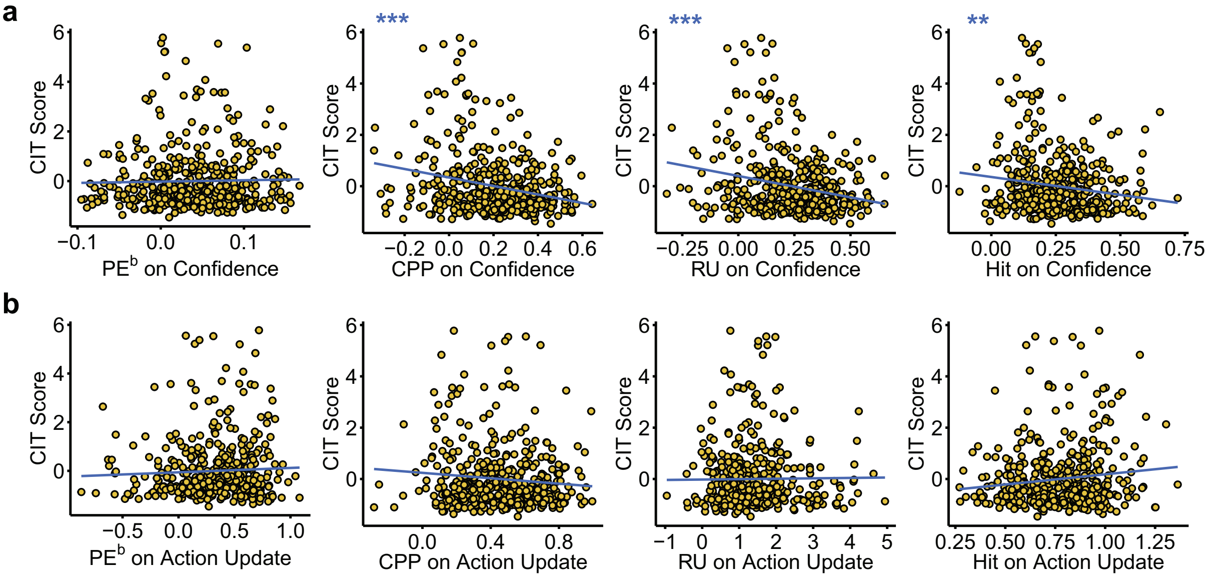

Supplementary Figure S5. Confidence level/action update was predicted by absolute model prediction error (PE^b), change-point probability (CPP), relative uncertainty (RU) and hit/miss categorial regressor (Hit), controlled for IQ, age and gender. Coefficient estimates from the model were correlated with 'compulsive behaviour and intrusive thought' (CIT) severity.

(a) CIT was found to be associated with significantly diminished influence of CPP, RU and Hit on z-scored confidence. PE^b, CPP and RU on confidence coefficients are inverted to illustrate direction of effects. PE^b: $r_s = 0.003$, $p = 1.00$; CPP: $r_s = -0.19$, $p < 0.001$; RU: $r_s = -0.17$, $p < 0.001$; Hit: $r_s = -0.15$, $p = 0.004$.

(b) In contrast, CIT was found not linked to changes in the influence of any of model parameters on action update. For plotting purposes, we show the association of parameter and compulsivity without controlling for AD and SW. PE^b: $r_s = 0.05$, $p = 0.99$; CPP: $r_s = -0.10$, $p = 0.14$; RU: $r_s = 0.01$, $p = 1.00$; Hit: $r_s = 0.09$, $p = 0.17$.

Circles represent coefficients of individual participants for model parameters from a basic mixed model of confidence/action update ~ regressors*demographics + (1 + regressors|subject) (x-axis), against their CIT score (y-axis) (see Methods). Hit on action update coefficients are inverted to illustrate direction of effects, such that CIT is linked to an increase influence of hits on action-updating (which is negative in direction). CI = Confidence interval. °p < 0.05, uncorrected, *p < 0.05, **p < 0.01, ***p < 0.001. Correlations are Spearman's rank correlations and results are Bonferroni corrected for multiple comparisons over the three dimensions. See also Figure 4.

Supplementary Table S3. Effects of 'compulsive behaviour and intrusive thought' (CIT) severity on Bayesian Model Parameters Coefficients on Action and Confidence, with heteroskedasticity-consistent standard errors. Model parameters on action or confidence coefficients were extracted from the basic mixed model action update/confidence ~ regressors*demographics + (1 + regressors|subject) and then regressed in a single linear model by all three psychiatric dimensions anxious-depression (AD), CIT and social withdrawal (SW) for each model parameter. Heteroskedasticity-consistent standard errors were estimated for each model by the *vcovHC* function from the *sandwich* package in R. Only CIT effects are reported here. In effect, results are similar to Supplementary Figure S5, but with all dimensions scores included in the same model. SE = standard Error, CI = confidence interval.

Predictor	***β (SE)***	***SE***	***t-value***	***p-value***
On Action				
PE[b]	*-0.0005*	*0.02*	*-0.03*	*0.98*
CPP	*-0.01*	*0.01*	*-0.79*	*0.43*
RU	*0.03*	*0.04*	*0.82*	*0.41*
Hit	*-0.01*	*0.01*	*-1.27*	*0.21*
On Confidence				
PE[b]	*-0.001*	*0.002*	*-0.58*	*0.57*
CPP	*0.04*	*0.007*	*6.07*	*< 0.001 ****
RU	*0.04*	*0.007*	*6.36*	*< 0.001 ****
Hit	*-0.23*	*0.006*	*-3.95*	*< 0.001 ****

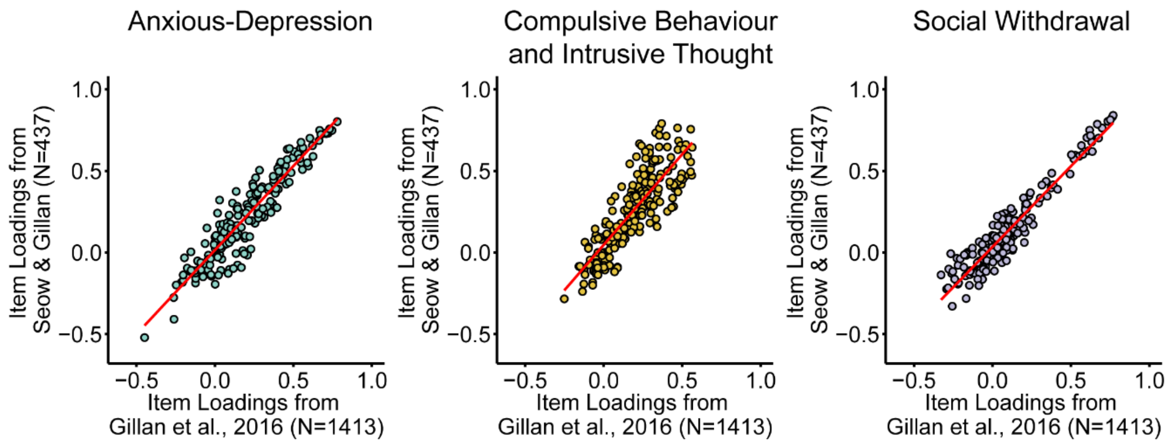

Supplementary Figure S6. Correlations between item loadings obtained from the factor analysis in Gillan *et al.* (2016) and the present study for each psychiatric symptom dimension. Questionnaire item loadings were highly correlated for all three dimensions (Anxious-depression: $r = 0.94$; Compulsive behaviour and intrusive thought: $r = 0.85$, Social withdrawal: $r = 0.95$), supporting the reproducibility of the psychiatric symptom dimensions.

Transdiagnostic symptom dimensions are reproducible. Transdiagnostic dimension scores (‘Anxious-depression’, ‘Compulsive behaviour and intrusive thoughts’, ‘Social withdrawal’) in the present study were derived from weights obtained from a prior larger study (N = 1413)[5]. This 3-factor structure was previously reproduced in a smaller independent sample (N = 497)[6], and here we again replicated similar psychiatric dimensions with our current data (N = 437) with the factor analysis (Supplementary Figure S6). For further details of the factor analysis methodology, see Gillan *et al.*[5].

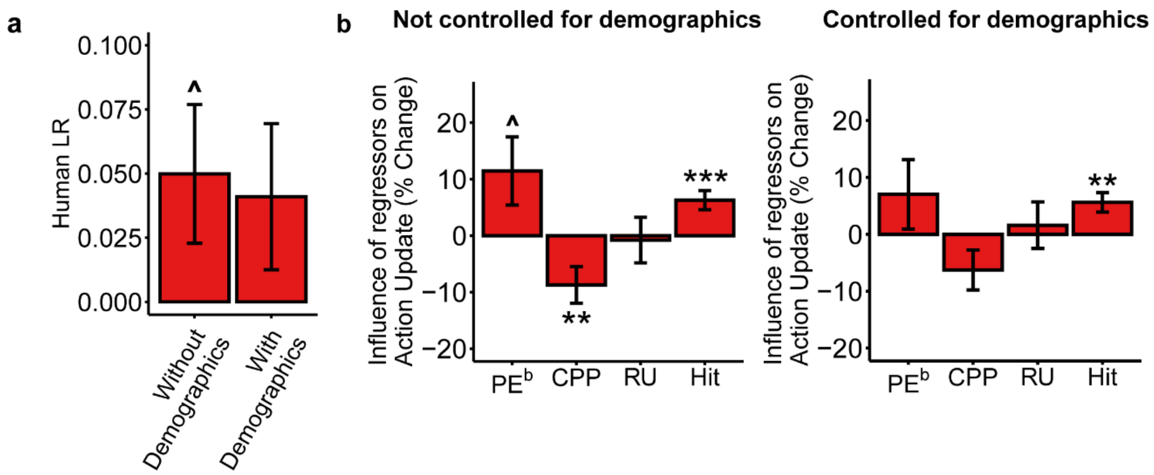

Supplementary Figure S7. Regression analyses of **(a)** human learning rate (ratio of bucket movement and task prediction error) and **(b)** action adjustments in OCD, in a model that controlled for age, IQ and gender and in a model that did not. Error bars denote standard errors. The Y-axes indicate the change/percentage change in dependent variable as a function of 1 standard deviation of OCD symptom scores. $^{\wedge}p < 0.07$, $^{**}p < 0.01$, $^{***}p < 0.001$. Results are not Bonferroni corrected for multiple comparisons.

Action updating effects in OCD with/without controlling for demographics. Vaghi *et al.*[1] reported that OCD patients exhibited a higher mean learning rate and that their action updates were more strongly influenced by recent information (PE^b) and less to large unexpected environmental changes (CPP). In the course of exploring the source of this discrepancy with our data, we found that when we repeated our analysis without controlling for age, gender and IQ, some of their effects were recovered here. OCD symptoms were associated with changes in learning and sensitivity to both PE^b and CPP in action updating. Specifically, LR^h (human learning rate) ($\beta = 0.05$, $SE = 0.03$, 95% CI [-0.003, 0.10], $p = 0.07$, uncorr.) and the influence of PE^b on action showed a trend towards a positive association with OCD symptoms ($\beta = 0.04$, $SE = 0.02$, 95% CI [-0.001, 0.07], $p = 0.06$, uncorr.) and the influence of CPP on action showed a negative association with OCD symptoms ($\beta = -0.04$, $SE = 0.02$, 95% CI [-0.07, -0.01], $p = 0.007$, uncorr.). These discrepancies suggest that demographic characteristics perhaps partially explain the pattern of action updating effects observed in the prior patient study (Supplementary Figure S7).

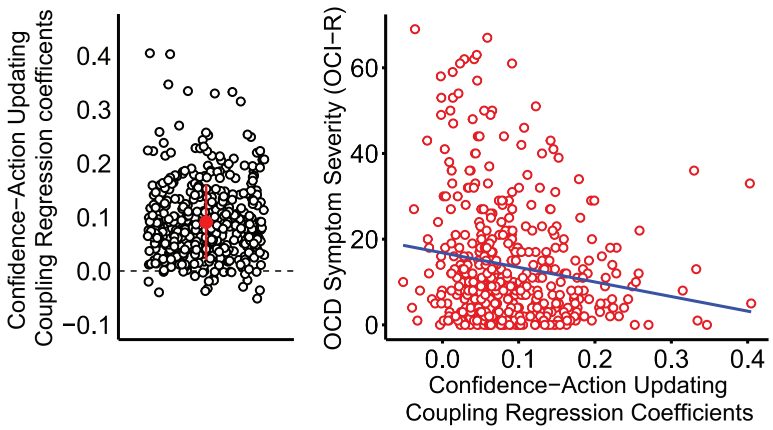

Supplementary Figure S8. Regression model where confidence updating was predicted by action updating. Dots represent coefficient estimates for individual participants. Red marker indicates mean and SD. These coefficients were correlated with OCD symptom severity, where confidence-action updating coupling was observed to decrease with increasing OCD symptom severity (r = -0.18, p < 0.001).

Action-confidence decoupling analysis. Although this has no bearing on our results (or theirs), we note that Vaghi *et al.*[1] defined action-confidence coupling slightly differently to how we chose to define it in the present paper – they used confidence *updating* (i.e. absolute difference between z-scored confidence from trial t and t-1), instead of the reported confidence level on trial t. We suggest that z-scored confidence ratings (rather than their change from trial to trial) are more appropriate because this accounts better for instances where a person has several relatively large PEs in a row (as they figure out where to place the bucket), and should thus not rationally 'change' their confidence rating in response to these PEs, but maintain it at a low level. Although we flag this for the interested reader, we underscore that the two measures are correlated and indeed when we use their definition, we similarly show that self-reported OCD symptom severity predicts confidence-action updating decoupling (r = -0.17, t = -3.58, 95% CI [-0.26, -0.07], p < 0.001, Supplementary Figure S8).

Supplemental References

1. Vaghi, M. M. *et al.* Compulsivity reveals a novel dissociation between action and confidence. *Neuron* **96**, 348–354 (2017).
2. McGuire, J. T., Nassar, M. R., Gold, J. I. & Kable, J. W. Functionally dissociable influences on learning rate in a dynamic environment. *Neuron* **84**, 870–881 (2014).
3. Nassar, M. R., Wilson, R. C., Heasly, B. & Gold, J. I. An approximately Bayesian delta-rule model explains the dynamics of belief updating in a changing environment. *J. Neurosci.* **30**, 12366–12378 (2010).
4. Nassar, M. R. *et al.* Age differences in learning emerge from an insufficient representation of uncertainty in older adults. *Nat. Commun.* **7**, 1–13 (2016).
5. Gillan, C. M., Kosinski, M., Whelan, R., Phelps, E. A. & Daw, N. D. Characterizing a psychiatric symptom dimension related to deficits in goal-directed control. *Elife* **5**, e11305 (2016).
6. Rouault, M., Seow, T., Gillan, C. M. & Fleming, S. M. Psychiatric symptom dimensions are associated with dissociable shifts in metacognition but not task performance. *Biol. Psychiatry* **84**, 443–451 (2018).
